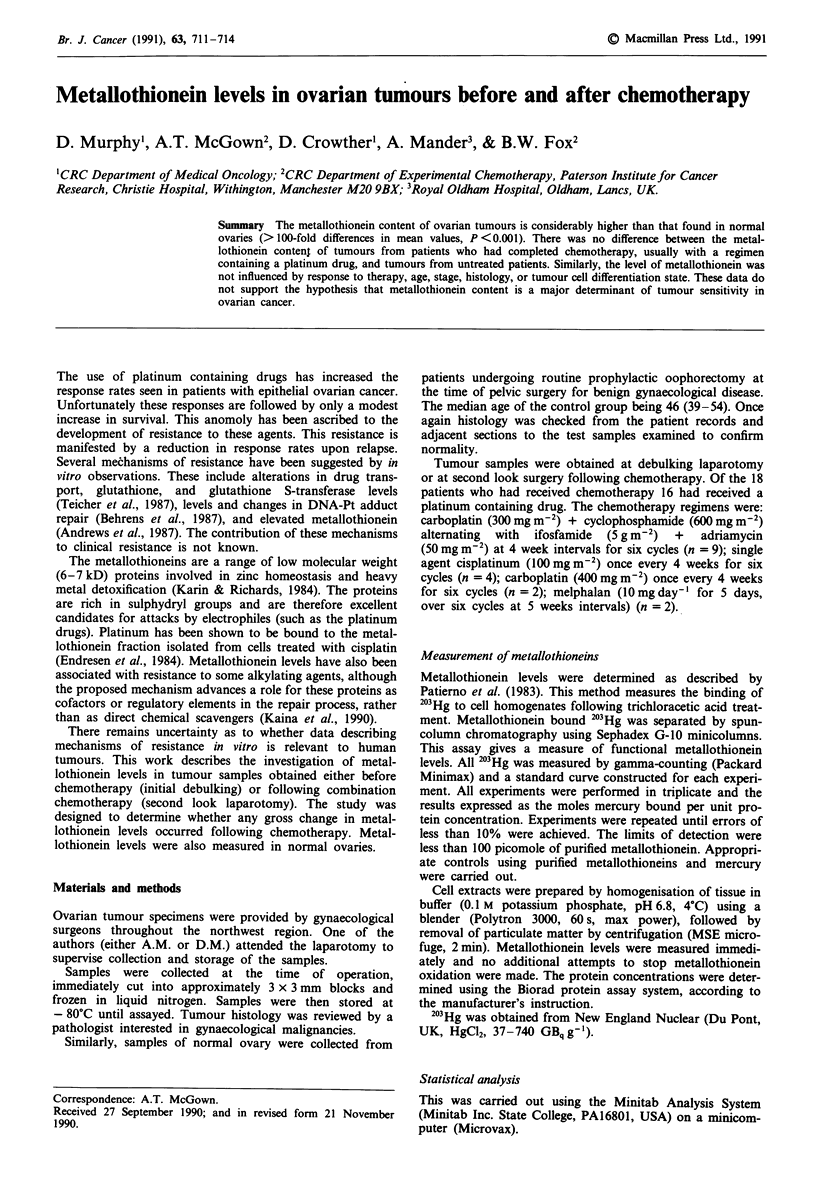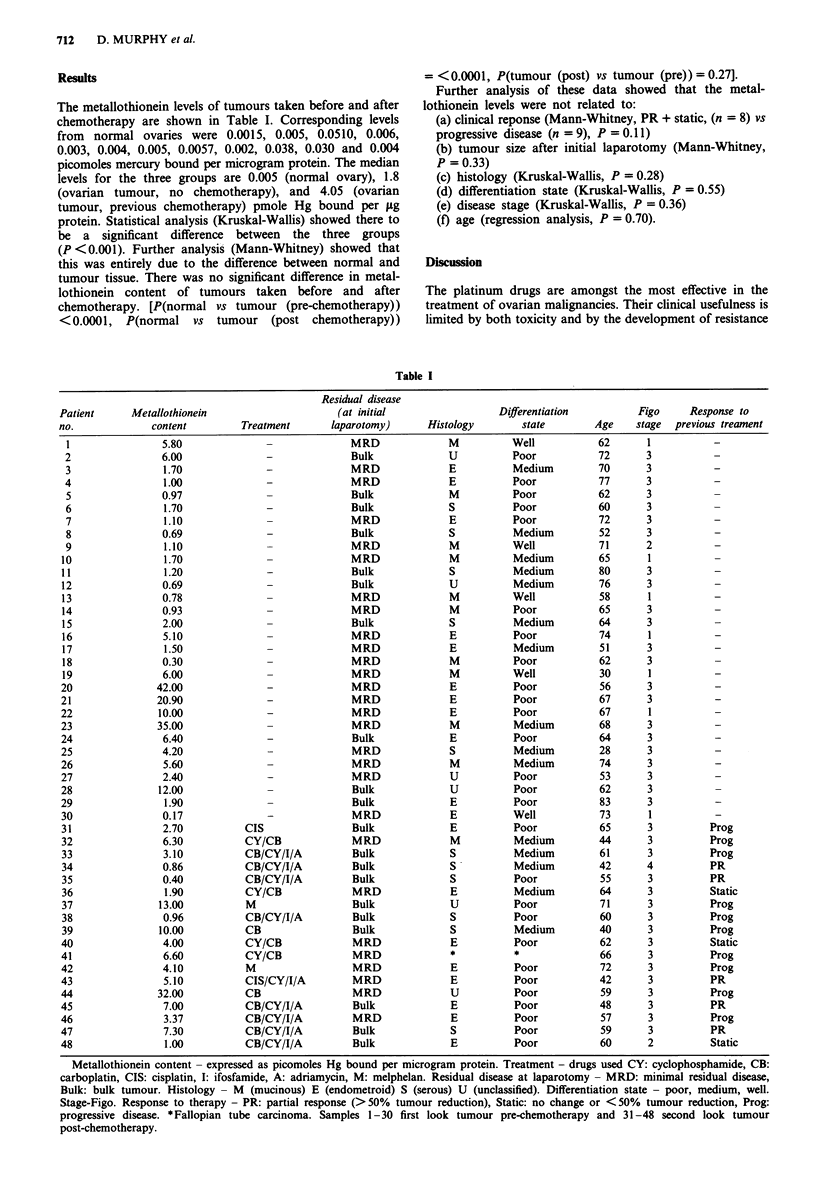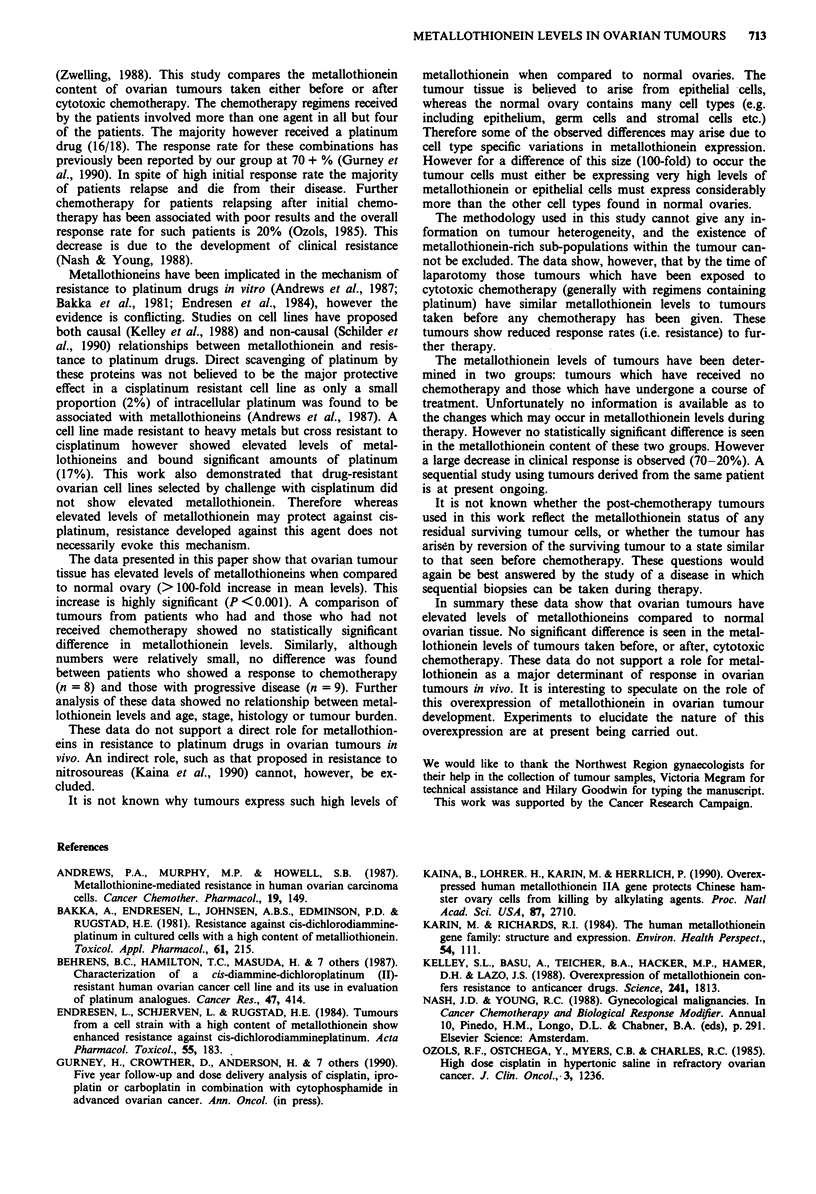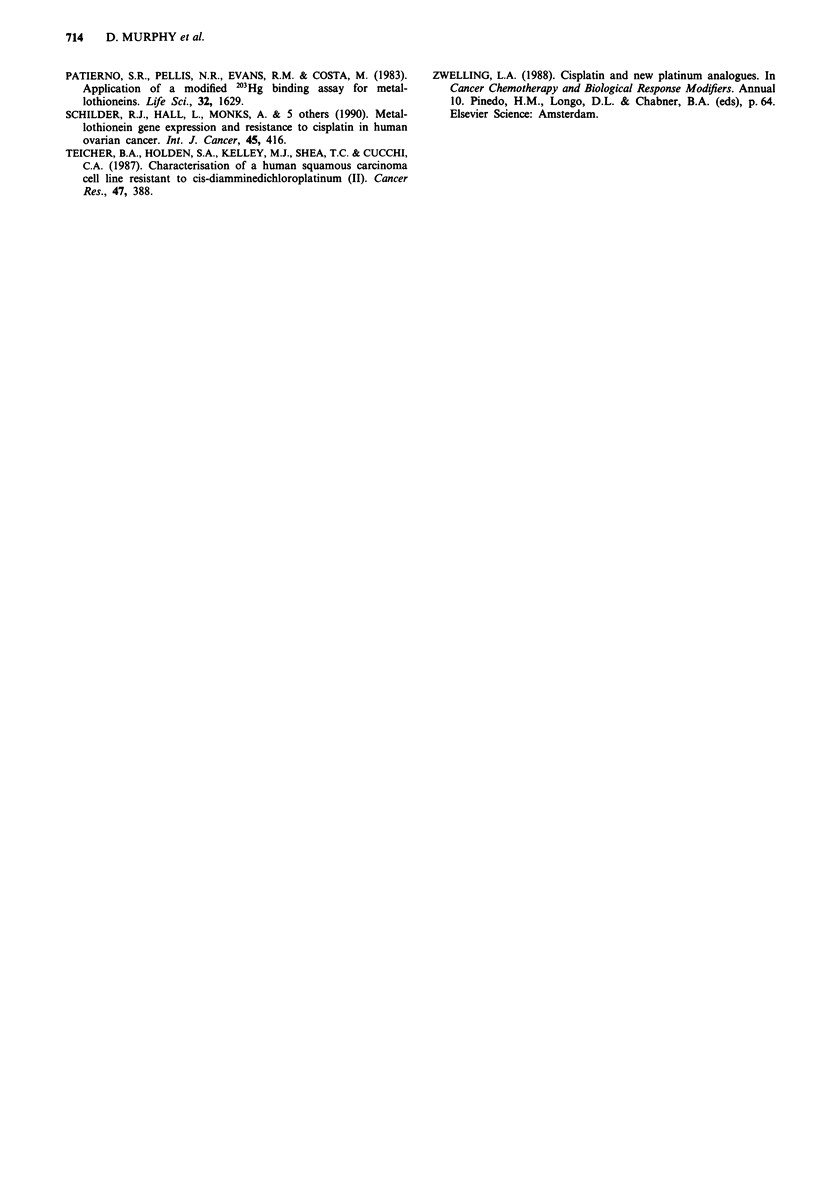# Metallothionein levels in ovarian tumours before and after chemotherapy.

**DOI:** 10.1038/bjc.1991.160

**Published:** 1991-05

**Authors:** D. Murphy, A. T. McGown, D. Crowther, A. Mander, B. W. Fox

**Affiliations:** CRC Department of Medical Oncology, Paterson Institute for Cancer Research, Christie Hospital, Withington, Manchester, UK.

## Abstract

The metallothionein content of ovarian tumours is considerably higher than that found in normal ovaries (greater than 100-fold differences in mean values, P less than 0.001). There was no difference between the metallothionein content of tumours from patients who had completed chemotherapy, usually with a regimen containing a platinum drug, and tumours from untreated patients. Similarly, the level of metallothionein was not influenced by response to therapy, age, stage, histology, or tumour cell differentiation state. These data do not support the hypothesis that metallothionein content is a major determinant of tumour sensitivity in ovarian cancer.


Br. J. Cancer (1991), 63, 711-714 © Macmillan Press Ltd., 1991

### Metallothionein levels in ovarian tumours before and after chemotherapy

D. Murphy[1], A.T. McGown[2], D. Crowther[1], A. Mander[3], & B.W. Fox[2]

[1]*CRC Department of Medical Oncology;* [2]*CRC Department of Experimental Chemotherapy, Paterson Institute for Cancer Research, Christie Hospital, Withington, Manchester M20 9BX;* [3]*Royal Oldham Hospital, Oldham, Lancs, UK.*

**Summary** The metallothionein content of ovarian tumours is considerably higher than that found in normal ovaries (> 100-fold differences in mean values, $P < 0.001$). There was no difference between the metallothionein content of tumours from patients who had completed chemotherapy, usually with a regimen containing a platinum drug, and tumours from untreated patients. Similarly, the level of metallothionein was not influenced by response to therapy, age, stage, histology, or tumour cell differentiation state. These data do not support the hypothesis that metallothionein content is a major determinant of tumour sensitivity in ovarian cancer.

The use of platinum containing drugs has increased the response rates seen in patients with epithelial ovarian cancer. Unfortunately these responses are followed by only a modest increase in survival. This anomoly has been ascribed to the development of resistance to these agents. This resistance is manifested by a reduction in response rates upon relapse. Several mechanisms of resistance have been suggested by *in vitro* observations. These include alterations in drug transport, glutathione, and glutathione S-transferase levels (Teicher *et al.*, 1987), levels and changes in DNA-Pt adduct repair (Behrens *et al.*, 1987), and elevated metallothionein (Andrews *et al.*, 1987). The contribution of these mechanisms to clinical resistance is not known.

The metallothioneins are a range of low molecular weight (6-7 kD) proteins involved in zinc homeostasis and heavy metal detoxification (Karin & Richards, 1984). The proteins are rich in sulphydryl groups and are therefore excellent candidates for attacks by electrophiles (such as the platinum drugs). Platinum has been shown to be bound to the metallothionein fraction isolated from cells treated with cisplatin (Endresen *et al.*, 1984). Metallothionein levels have also been associated with resistance to some alkylating agents, although the proposed mechanism advances a role for these proteins as cofactors or regulatory elements in the repair process, rather than as direct chemical scavengers (Kaina *et al.*, 1990).

There remains uncertainty as to whether data describing mechanisms of resistance *in vitro* is relevant to human tumours. This work describes the investigation of metallothionein levels in tumour samples obtained either before chemotherapy (initial debulking) or following combination chemotherapy (second look laparotomy). The study was designed to determine whether any gross change in metallothionein levels occurred following chemotherapy. Metallothionein levels were also measured in normal ovaries.

### Materials and methods

Ovarian tumour specimens were provided by gynaecological surgeons throughout the northwest region. One of the authors (either A.M. or D.M.) attended the laparotomy to supervise collection and storage of the samples.

Samples were collected at the time of operation, immediately cut into approximately 3 × 3 mm blocks and frozen in liquid nitrogen. Samples were then stored at − 80°C until assayed. Tumour histology was reviewed by a pathologist interested in gynaecological malignancies.

Similarly, samples of normal ovary were collected from patients undergoing routine prophylactic oophorectomy at the time of pelvic surgery for benign gynaecological disease. The median age of the control group being 46 (39-54). Once again histology was checked from the patient records and adjacent sections to the test samples examined to confirm normality.

Tumour samples were obtained at debulking laparotomy or at second look surgery following chemotherapy. Of the 18 patients who had received chemotherapy 16 had received a platinum containing drug. The chemotherapy regimens were: carboplatin (300 mg m$^{-2}$) + cyclophosphamide (600 mg m$^{-2}$) alternating with ifosfamide (5 g m$^{-2}$) + adriamycin (50 mg m$^{-2}$) at 4 week intervals for six cycles ($n = 9$); single agent cisplatinum (100 mg m$^{-2}$) once every 4 weeks for six cycles ($n = 4$); carboplatin (400 mg m$^{-2}$) once every 4 weeks for six cycles ($n = 2$); melphalan (10 mg day$^{-1}$ for 5 days, over six cycles at 5 weeks intervals) ($n = 2$).

### *Measurement of metallothioneins*

Metallothionein levels were determined as described by Patierno *et al.* (1983). This method measures the binding of $^{203}$Hg to cell homogenates following trichloracetic acid treatment. Metallothionein bound $^{203}$Hg was separated by spun-column chromatography using Sephadex G-10 minicolumns. This assay gives a measure of functional metallothionein levels. All $^{203}$Hg was measured by gamma-counting (Packard Minimax) and a standard curve constructed for each experiment. All experiments were performed in triplicate and the results expressed as the moles mercury bound per unit protein concentration. Experiments were repeated until errors of less than 10% were achieved. The limits of detection were less than 100 picomole of purified metallothionein. Appropriate controls using purified metallothioneins and mercury were carried out.

Cell extracts were prepared by homogenisation of tissue in buffer (0.1 M potassium phosphate, pH 6.8, 4°C) using a blender (Polytron 3000, 60 s, max power), followed by removal of particulate matter by centrifugation (MSE microfuge, 2 min). Metallothionein levels were measured immediately and no additional attempts to stop metallothionein oxidation were made. The protein concentrations were determined using the Biorad protein assay system, according to the manufacturer's instruction.

$^{203}$Hg was obtained from New England Nuclear (Du Pont, UK, $HgCl_2$, 37-740 $GB_q\,g^{-1}$).

### *Statistical analysis*

This was carried out using the Minitab Analysis System (Minitab Inc. State College, PA16801, USA) on a minicomputer (Microvax).

Correspondence: A.T. McGown.
Received 27 September 1990; and in revised form 21 November 1990.

### Results

The metallothionein levels of tumours taken before and after chemotherapy are shown in Table I. Corresponding levels from normal ovaries were 0.0015, 0.005, 0.0510, 0.006, 0.003, 0.004, 0.005, 0.0057, 0.002, 0.038, 0.030 and 0.004 picomoles mercury bound per microgram protein. The median levels for the three groups are 0.005 (normal ovary), 1.8 (ovarian tumour, no chemotherapy), and 4.05 (ovarian tumour, previous chemotherapy) pmole Hg bound per μg protein. Statistical analysis (Kruskal-Wallis) showed there to be a significant difference between the three groups ($P < 0.001$). Further analysis (Mann-Whitney) showed that this was entirely due to the difference between normal and tumour tissue. There was no significant difference in metallothionein content of tumours taken before and after chemotherapy. [$P$(normal *vs* tumour (pre-chemotherapy)) $< 0.0001$, $P$(normal *vs* tumour (post chemotherapy)) $= < 0.0001$, $P$(tumour (post) *vs* tumour (pre)) $= 0.27$].

Further analysis of these data showed that the metallothionein levels were not related to:

(a) clinical reponse (Mann-Whitney, PR + static, ($n = 8$) *vs* progressive disease ($n = 9$), $P = 0.11$)
(b) tumour size after initial laparotomy (Mann-Whitney, $P = 0.33$)
(c) histology (Kruskal-Wallis, $P = 0.28$)
(d) differentiation state (Kruskal-Wallis, $P = 0.55$)
(e) disease stage (Kruskal-Wallis, $P = 0.36$)
(f) age (regression analysis, $P = 0.70$).

### Discussion

The platinum drugs are amongst the most effective in the treatment of ovarian malignancies. Their clinical usefulness is limited by both toxicity and by the development of resistance

**Table I**

| *Patient no.* | *Metallothionein content* | *Treatment* | *Residual disease (at initial laparotomy)* | *Histology* | *Differentiation state* | *Age* | *Figo stage* | *Response to previous treament* |
|---|---|---|---|---|---|---|---|---|
| 1 | 5.80 | – | MRD | M | Well | 62 | 1 | – |
| 2 | 6.00 | – | Bulk | U | Poor | 72 | 3 | – |
| 3 | 1.70 | – | MRD | E | Medium | 70 | 3 | – |
| 4 | 1.00 | – | MRD | E | Poor | 77 | 3 | – |
| 5 | 0.97 | – | Bulk | M | Poor | 62 | 3 | – |
| 6 | 1.70 | – | Bulk | S | Poor | 60 | 3 | – |
| 7 | 1.10 | – | MRD | E | Poor | 72 | 3 | – |
| 8 | 0.69 | – | Bulk | S | Medium | 52 | 3 | – |
| 9 | 1.10 | – | MRD | M | Well | 71 | 2 | – |
| 10 | 1.70 | – | MRD | M | Medium | 65 | 1 | – |
| 11 | 1.20 | – | Bulk | S | Medium | 80 | 3 | – |
| 12 | 0.69 | – | Bulk | U | Medium | 76 | 3 | – |
| 13 | 0.78 | – | MRD | M | Well | 58 | 1 | – |
| 14 | 0.93 | – | MRD | M | Poor | 65 | 3 | – |
| 15 | 2.00 | – | Bulk | S | Medium | 64 | 3 | – |
| 16 | 5.10 | – | MRD | E | Poor | 74 | 1 | – |
| 17 | 1.50 | – | MRD | E | Medium | 51 | 3 | – |
| 18 | 0.30 | – | MRD | M | Poor | 62 | 3 | – |
| 19 | 6.00 | – | MRD | M | Well | 30 | 1 | – |
| 20 | 42.00 | – | MRD | E | Poor | 56 | 3 | – |
| 21 | 20.90 | – | MRD | E | Poor | 67 | 3 | – |
| 22 | 10.00 | – | MRD | E | Poor | 67 | 1 | – |
| 23 | 35.00 | – | MRD | M | Medium | 68 | 3 | – |
| 24 | 6.40 | – | Bulk | E | Poor | 64 | 3 | – |
| 25 | 4.20 | – | MRD | S | Medium | 28 | 3 | – |
| 26 | 5.60 | – | MRD | M | Medium | 74 | 3 | – |
| 27 | 2.40 | – | MRD | U | Poor | 53 | 3 | – |
| 28 | 12.00 | – | Bulk | U | Poor | 62 | 3 | – |
| 29 | 1.90 | – | Bulk | E | Poor | 83 | 3 | – |
| 30 | 0.17 | – | MRD | E | Well | 73 | 1 | – |
| 31 | 2.70 | CIS | Bulk | E | Poor | 65 | 3 | Prog |
| 32 | 6.30 | CY/CB | MRD | M | Medium | 44 | 3 | Prog |
| 33 | 3.10 | CB/CY/I/A | Bulk | S | Medium | 61 | 3 | Prog |
| 34 | 0.86 | CB/CY/I/A | Bulk | S | Medium | 42 | 4 | PR |
| 35 | 0.40 | CB/CY/I/A | Bulk | S | Poor | 55 | 3 | PR |
| 36 | 1.90 | CY/CB | MRD | E | Medium | 64 | 3 | Static |
| 37 | 13.00 | M | Bulk | U | Poor | 71 | 3 | Prog |
| 38 | 0.96 | CB/CY/I/A | Bulk | S | Poor | 60 | 3 | Prog |
| 39 | 10.00 | CB | Bulk | S | Medium | 40 | 3 | Prog |
| 40 | 4.00 | CY/CB | MRD | E | Poor | 62 | 3 | Static |
| 41 | 6.60 | CY/CB | MRD | * | * | 66 | 3 | Prog |
| 42 | 4.10 | M | MRD | E | Poor | 72 | 3 | Prog |
| 43 | 5.10 | CIS/CY/I/A | MRD | E | Poor | 42 | 3 | PR |
| 44 | 32.00 | CB | MRD | U | Poor | 59 | 3 | Prog |
| 45 | 7.00 | CB/CY/I/A | Bulk | E | Poor | 48 | 3 | PR |
| 46 | 3.37 | CB/CY/I/A | MRD | E | Poor | 57 | 3 | Prog |
| 47 | 7.30 | CB/CY/I/A | Bulk | S | Poor | 59 | 3 | PR |
| 48 | 1.00 | CB/CY/I/A | Bulk | E | Poor | 60 | 2 | Static |

Metallothionein content – expressed as picomoles Hg bound per microgram protein. Treatment – drugs used CY: cyclophosphamide, CB: carboplatin, CIS: cisplatin, I: ifosfamide, A: adriamycin, M: melphelan. Residual disease at laparotomy – MRD: minimal residual disease, Bulk: bulk tumour. Histology – M (mucinous) E (endometroid) S (serous) U (unclassified). Differentiation state – poor, medium, well. Stage-Figo. Response to therapy – PR: partial response (>50% tumour reduction), Static: no change or <50% tumour reduction, Prog: progressive disease. *Fallopian tube carcinoma. Samples 1–30 first look tumour pre-chemotherapy and 31–48 second look tumour post-chemotherapy.

(Zwelling, 1988). This study compares the metallothionein content of ovarian tumours taken either before or after cytotoxic chemotherapy. The chemotherapy regimens received by the patients involved more than one agent in all but four of the patients. The majority however received a platinum drug (16/18). The response rate for these combinations has previously been reported by our group at 70 + % (Gurney *et al.*, 1990). In spite of high initial response rate the majority of patients relapse and die from their disease. Further chemotherapy for patients relapsing after initial chemotherapy has been associated with poor results and the overall response rate for such patients is 20% (Ozols, 1985). This decrease is due to the development of clinical resistance (Nash & Young, 1988).

Metallothioneins have been implicated in the mechanism of resistance to platinum drugs *in vitro* (Andrews *et al.*, 1987; Bakka *et al.*, 1981; Endresen *et al.*, 1984), however the evidence is conflicting. Studies on cell lines have proposed both causal (Kelley *et al.*, 1988) and non-causal (Schilder *et al.*, 1990) relationships between metallothionein and resistance to platinum drugs. Direct scavenging of platinum by these proteins was not believed to be the major protective effect in a cisplatinum resistant cell line as only a small proportion (2%) of intracellular platinum was found to be associated with metallothioneins (Andrews *et al.*, 1987). A cell line made resistant to heavy metals but cross resistant to cisplatinum however showed elevated levels of metallothioneins and bound significant amounts of platinum (17%). This work also demonstrated that drug-resistant ovarian cell lines selected by challenge with cisplatinum did not show elevated metallothionein. Therefore whereas elevated levels of metallothionein may protect against cisplatinum, resistance developed against this agent does not necessarily evoke this mechanism.

The data presented in this paper show that ovarian tumour tissue has elevated levels of metallothioneins when compared to normal ovary (> 100-fold increase in mean levels). This increase is highly significant ($P < 0.001$). A comparison of tumours from patients who had and those who had not received chemotherapy showed no statistically significant difference in metallothionein levels. Similarly, although numbers were relatively small, no difference was found between patients who showed a response to chemotherapy ($n = 8$) and those with progressive disease ($n = 9$). Further analysis of these data showed no relationship between metallothionein levels and age, stage, histology or tumour burden.

These data do not support a direct role for metallothioneins in resistance to platinum drugs in ovarian tumours *in vivo*. An indirect role, such as that proposed in resistance to nitrosoureas (Kaina *et al.*, 1990) cannot, however, be excluded.

It is not known why tumours express such high levels of metallothionein when compared to normal ovaries. The tumour tissue is believed to arise from epithelial cells, whereas the normal ovary contains many cell types (e.g. including epithelium, germ cells and stromal cells etc.) Therefore some of the observed differences may arise due to cell type specific variations in metallothionein expression. However for a difference of this size (100-fold) to occur the tumour cells must either be expressing very high levels of metallothionein or epithelial cells must express considerably more than the other cell types found in normal ovaries.

The methodology used in this study cannot give any information on tumour heterogeneity, and the existence of metallothionein-rich sub-populations within the tumour cannot be excluded. The data show, however, that by the time of laparotomy those tumours which have been exposed to cytotoxic chemotherapy (generally with regimens containing platinum) have similar metallothionein levels to tumours taken before any chemotherapy has been given. These tumours show reduced response rates (i.e. resistance) to further therapy.

The metallothionein levels of tumours have been determined in two groups: tumours which have received no chemotherapy and those which have undergone a course of treatment. Unfortunately no information is available as to the changes which may occur in metallothionein levels during therapy. However no statistically significant difference is seen in the metallothionein content of these two groups. However a large decrease in clinical response is observed (70–20%). A sequential study using tumours derived from the same patient is at present ongoing.

It is not known whether the post-chemotherapy tumours used in this work reflect the metallothionein status of any residual surviving tumour cells, or whether the tumour has arisen by reversion of the surviving tumour to a state similar to that seen before chemotherapy. These questions would again be best answered by the study of a disease in which sequential biopsies can be taken during therapy.

In summary these data show that ovarian tumours have elevated levels of metallothioneins compared to normal ovarian tissue. No significant difference is seen in the metallothionein levels of tumours taken before, or after, cytotoxic chemotherapy. These data do not support a role for metallothionein as a major determinant of response in ovarian tumours *in vivo*. It is interesting to speculate on the role of this overexpression of metallothionein in ovarian tumour development. Experiments to elucidate the nature of this overexpression are at present being carried out.

We would like to thank the Northwest Region gynaecologists for their help in the collection of tumour samples, Victoria Megram for technical assistance and Hilary Goodwin for typing the manuscript.
This work was supported by the Cancer Research Campaign.

### References

ANDREWS, P.A., MURPHY, M.P. & HOWELL, S.B. (1987). Metallothionine-mediated resistance in human ovarian carcinoma cells. *Cancer Chemother. Pharmacol.*, **19**, 149.

BAKKA, A., ENDRESEN, L., JOHNSEN, A.B.S., EDMINSON, P.D. & RUGSTAD, H.E. (1981). Resistance against cis-dichlorodiammine-platinum in cultured cells with a high content of metalliothionein. *Toxicol. Appl. Pharmacol.*, **61**, 215.

BEHRENS, B.C., HAMILTON, T.C., MASUDA, H. & 7 others (1987). Characterization of a *cis*-diammine-dichloroplatinum (II)-resistant human ovarian cancer cell line and its use in evaluation of platinum analogues. *Cancer Res.*, **47**, 414.

ENDRESEN, L., SCHJERVEN, L. & RUGSTAD, H.E. (1984). Tumours from a cell strain with a high content of metallothionein show enhanced resistance against cis-dichlorodiammineplatinum. *Acta Pharmacol. Toxicol.*, **55**, 183.

GURNEY, H., CROWTHER, D., ANDERSON, H. & 7 others (1990). Five year follow-up and dose delivery analysis of cisplatin, iproplatin or carboplatin in combination with cytophosphamide in advanced ovarian cancer. *Ann. Oncol.* (in press).

KAINA, B., LOHRER. H., KARIN, M. & HERRLICH, P. (1990). Overexpressed human metallothionein IIA gene protects Chinese hamster ovary cells from killing by alkylating agents. *Proc. Natl Acad. Sci. USA*, **87**, 2710.

KARIN, M. & RICHARDS, R.I. (1984). The human metallothionein gene family: structure and expression. *Environ. Health Perspect.*, **54**, 111.

KELLEY, S.L., BASU, A., TEICHER, B.A., HACKER, M.P., HAMER, D.H. & LAZO, J.S. (1988). Overexpression of metallothionein confers resistance to anticancer drugs. *Science*, **241**, 1813.

NASH, J.D. & YOUNG, R.C. (1988). Gynecological malignancies. In *Cancer Chemotherapy and Biological Response Modifier*. Annual 10, Pinedo, H.M., Longo, D.L. & Chabner, B.A. (eds), p. 291. Elsevier Science: Amsterdam.

OZOLS, R.F., OSTCHEGA, Y., MYERS, C.B. & CHARLES, R.C. (1985). High dose cisplatin in hypertonic saline in refractory ovarian cancer. *J. Clin. Oncol.*, **3**, 1236.

PATIERNO, S.R., PELLIS, N.R., EVANS, R.M. & COSTA, M. (1983). Application of a modified $^{203}Hg$ binding assay for metallothioneins. *Life Sci.*, **32**, 1629.

SCHILDER, R.J., HALL, L., MONKS, A. & 5 others (1990). Metallothionein gene expression and resistance to cisplatin in human ovarian cancer. *Int. J. Cancer*, **45**, 416.

TEICHER, B.A., HOLDEN, S.A., KELLEY, M.J., SHEA, T.C. & CUCCHI, C.A. (1987). Characterisation of a human squamous carcinoma cell line resistant to cis-diamminedichloroplatinum (II). *Cancer Res.*, **47**, 388.

ZWELLING, L.A. (1988). Cisplatin and new platinum analogues. In *Cancer Chemotherapy and Biological Response Modifiers*. Annual 10. Pinedo, H.M., Longo, D.L. & Chabner, B.A. (eds), p. 64. Elsevier Science: Amsterdam.